# Investigation of Mechanical Properties of Ultra-High-Performance Polyethylene-Fiber-Reinforced Recycled-Brick-Aggregate Concrete

**DOI:** 10.3390/polym15234573

**Published:** 2023-11-29

**Authors:** Yongcheng Ji, Zhiyang Pei

**Affiliations:** School of Civil Engineering and Transportation, Northeast Forestry University, Harbin 150040, China; sirpzy@163.com

**Keywords:** ultra-high-molecular-weight polyethylene fiber, recycled brick aggregate, durability, response-surface methodology, regression model, multiobjective optimization

## Abstract

The utilization of ultra-high-molecular-weight polyethylene fibers (UHMWPEFs) to enhance recycled-brick-aggregate concrete represents an efficacious approach for ameliorating the concrete’s performance. This investigation addresses the influences of recycled-brick aggregates (RAs) and UHMWPEFs on the concrete’s slump, shrinkage, flexural strength, resistance to chloride-ion ingress, and freeze–thaw durability. The mechanisms through which UHMWPEFs ameliorate the performance of the recycled-brick-aggregate concrete were elucidated at both the micro and macroscopic levels. The findings underscore that the three-dimensional network structure established by the UHMWPEFs, while resulting in a reduction in the concrete slump, substantially enhances the concrete’s mechanical properties and durability. A regression model for the multifaceted performance of the UHMWPEF-reinforced recycled-brick-aggregate concrete (F-RAC) was formulated by employing response-surface methodology, and the model’s reliability was confirmed through variance analysis. The interactive effects of the RA and UHMWPEFs on the concrete were analyzed through a combined approach involving response-surface analysis and contour plots. Subsequently, a multiobjective optimization was conducted for the F-RAC performance, yielding the optimal proportions of RA and UHMWPEFs. It was determined that the optimal performance across the dimensions of the shrinkage resistance, flexural strength, chloride-ion resistance, and freeze–thaw durability of the F-RAC could be simultaneously achieved when the substitution rate of the RA was 14.02% and the admixture of the UHMWPEFs was 1.13%.

## 1. Introduction

**Figure 1:**
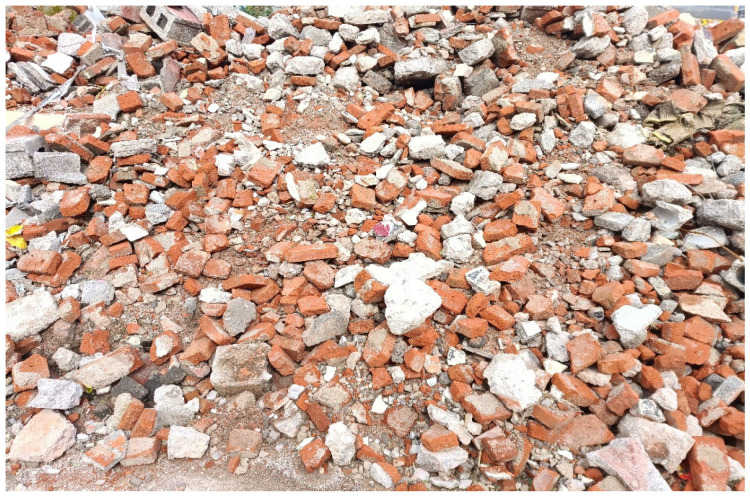
Waste bricks in construction waste.

Concrete presently stands as one of the most widely employed and abundantly consumed construction materials. Recent statistics have indicated a global annual consumption of approximately 200 billion metric tons of concrete [1]. Within concrete compositions, aggregates constitute approximately 60% to 75% of the overall volume [2]. Consequently, as the demand for concrete continues to surge, so does the requirement for aggregates. Simultaneously, the rapid urbanization process has led to the demolition of a substantial number of aged structures, consequently generating substantial volumes of construction debris. Among this architectural waste, waste bricks account for approximately 35–45% of the total volume [3], with a potential increase in rural regions where this ratio can escalate to 50–70%. Figure 1 illustrates a demolition site where discarded bricks are ubiquitous. The transformation of these discarded bricks into RA mitigates the reliance on natural aggregates (NAs) and contributes to the alleviation of environmental pollution issues associated with construction waste.

Crushed bricks were first extensively employed as aggregates in concrete in the post-World War II reconstruction era [4]. Rashid et al. [5] and Akhtaruzzaman et al. [6] produced recycled-brick-aggregate concretes using conventional concrete-making methods without adding any admixtures. Notably, these concretes exhibited a readily achievable compressive strength of 20 MPa. Debieb et al. [7] investigated the shrinkage performance of recycled concrete by varying the particle size of different brick aggregates. The findings indicated that an increase in the proportion of brick aggregates leads to an escalation in the shrinkage rate of the recycled concrete. Compared with conventional concrete, recycled-brick-aggregate concrete exhibits a slightly reduced ultimate load-bearing capacity and a heightened propensity for crack formation [8]. These shortcomings have the potential to curtail the service life of concrete structures.

To mitigate these shortcomings of recycled-brick-aggregate concrete, researchers have sought improvements through adjustments in the water-to-cement ratio, the utilization of CFRP wrapping, and the incorporation of fibers. Among these approaches, the addition of fibers has emerged as a highly effective and operationally feasible method. Islam et al. employed steel fibers measuring 50 mm in length and possessing a length-to-diameter ratio of 55.6 as the reinforcing material for recycled concrete [9]. The results indicated that the introduction of steel fibers leads to a significant enhancement in both the compressive and flexural strengths of recycled concrete, with increases of 10–15% and 40–60%, respectively. Notably, compared with control groups, concrete specimens containing steel fibers exhibit more significant strain at the point of failure. Although steel fibers can enhance the mechanical properties of concrete, they tend to reduce the compactness of the concrete matrix [10]. This affects the permeability and durability of the concrete. Furthermore, the incorporation of steel fibers into the concrete introduces magnetism, potentially yielding implications for specific specialized engineering applications. Elshazli et al. [11] investigated the impact of basalt fibers on the durability of recycled concrete. Their research revealed that as the volume fraction of the basalt fibers increases, the slump of the concrete decreases. In the presence of basalt fibers, both the mechanical properties and durability of the recycled concrete improve. However, the corrosion rate of the concrete increases with the increasing volume fraction of the basalt fibers. Małek et al. [12] manufactured recycled polypropylene fibers from plastic waste and incorporated them into concrete to enhance its mechanical properties. The research indicated that the inclusion of the recycled polypropylene fibers enhanced the flexural strength and ductility of the concrete, albeit with a comparatively modest improvement in the flexural strength. When the content of recycled polypropylene fibers in concrete reaches 1.0%, the compressive strength and flexural strength of the concrete increase by 39.4% and 162.4%, respectively. Nevertheless, the tensile strength and elastic modulus of polypropylene fibers are relatively low, limiting their reinforcing efficacy and applicability. Particularly for specific high-strength engineering requirements, the reinforcing effects of polypropylene fibers fail to meet design specifications.

In comparison to other commonly encountered fibers, UHMWPEF exhibits pronounced advantages. The molecular weight of polypropylene fiber is approximately 5.2 × 10^4^ g/mol [13], while that of polyvinyl alcohol fiber is approximately 7.8 × 10^4^ g/mol [14]. In contrast, the molecular weight of regular polyethylene is typically not higher than 2 × 10^5^ g/mol [15]. However, the molecular weight of UHMWPEF can easily exceed 3.1 × 10^6^ g/mol [15], and it can be even higher under different production processes. The high molecular weight of UHMWPEF imparts it with superior toughness and strength, enabling it to exhibit an exceptional tensile performance compared to other fibers [16]. It effectively ameliorates stress distribution within concrete, consequently enhancing the tensile strength of concrete. Osman et al. [17] investigated the influence of PVA fibers and UHMWPEF on concrete’s dynamic compression and tension properties. The results indicated that the enhancement effect of UHMWPEF on the mechanical and crack resistance properties of concrete surpassed that of PVA fibers. Compared to ordinary concrete, the addition of UHMWPEF increased the compressive strength of concrete by 23% and the ultimate strain by 17.5%. Gong et al. [18] studied the effect of UHMWPEF on the tensile properties of cement-based composite materials. The experimental results demonstrated the exceptional energy absorption capability of UHMWPEF. Under the influence of UHMWPEF, the tensile strength of concrete was significantly enhanced, and its toughness and crack control capability were also strengthened. To improve the tensile strength and ductility of concrete, Li et al. [19] incorporated UHMWPEF into concrete and conducted mechanical and explosion tests. The research revealed that adding UHMWPEF resulted in an improved flexural performance and energy absorption capacity of concrete. However, the compressive performance of concrete was less affected by UHMWPEF. Zhang et al. [20] investigated the influence of different volume fractions of UHMWPEF on concrete’s mechanical and permeability properties. The study pointed out that a volume fraction of 0.8% of UHMWPEF effectively enhanced the ductility of concrete, which is closely related to its high elastic modulus and tensile strength. Game et al. [21] applied UHMWPEF in 3D-printed concrete technology to produce ultra-high-performance cement composite materials. The research revealed that while UHMWPEF extended the setting time and flowability of the cement composite material, it also improved its shape stability. After 28 days of curing, the UHMWPEF-reinforced cement composite material exhibited a superior flexural strength and crack resistance compared to ordinary cement composite materials. Furthermore, UHMWPEF possesses outstanding wear, corrosion, and fatigue resistance compared to other fibers, which enables it to withstand the corrosion of chemical substances such as acids and alkalis without compromising its performance [22]. Additionally, the low density of UHMWPEF does not significantly increase the weight of concrete, which is crucial for large-span structures and those that require weight reduction.

Other scholars have predominantly emphasized the analysis of fiber impact on the mechanical properties of concrete; there needs to be more investigation into durability aspects. This research marks the inaugural application of UHMWPEF for the enhancement of recycled-brick-aggregate concrete, thus introducing novel avenues for enhancing the performance of such concrete. By manipulating the replacement ratio of RA instead of NA and varying the volume fraction of UHMWPEF, this research examines their influence on concrete slump, shrinkage, flexural strength, resistance to chloride ion ingress, and freeze–thaw durability. A dual perspective, encompassing both micro and macro levels, is employed to explore the multifaceted performance of concrete under the influence of RA while elucidating the reinforcement mechanisms of UHMWPEF. Furthermore, in this research, a regression model encompassing various aspects of performance in F-RAC was established using response-surface methodology. The reliability of the model was confirmed through variance analysis. By scrutinizing response-surface plots and contour graphs, the interactions between RA and UHMWPEF on concrete were elucidated. Lastly, three optimization objectives were posited, and the optimal proportions of RA and UHMWPEF were determined in conjunction with the regression model.

## 2. Materials and Methods

### 2.1. Materials

#### 2.1.1. Aggregate

**Figure 2:**
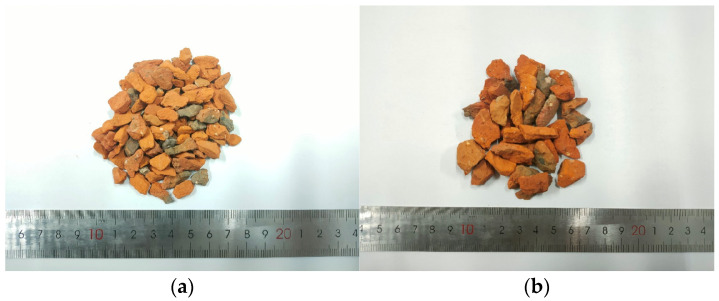
Recycled-brick aggregates: (**a**) Particle sizes ranging from 5 to 10 mm; (**b**) Particle sizes ranging from 10 to 20 mm.

The study employed clay red bricks as the primary construction material, carefully selected from discarded building materials. The RA is derived from these clay-red bricks subjected to a series of procedures, including crushing, sieving, cleansing, and drying. The chemical composition of RA is shown in Table 1. Following the guidelines of the Chinese standard GB/T 14685-2011 [23], RA underwent classification into two size fractions: 5–10 mm and 10–20 mm, as illustrated in Figure 2. NA consisted of limestone, purchased from Shou Xian building materials company in Harbin, China. The physical characteristics of both aggregate types are presented in Table 2.

#### 2.1.2. Ultra-High Molecular Weight Polyethylene Fiber

Table 3 presents the physical properties and morphology of UHMWPEF alongside three other fibers commonly employed for enhancing concrete performance. Due to their larger diameter, steel fibers tend to generate voids within the concrete, thereby diminishing concrete continuity and compactness. Basalt fibers, conversely, exhibit a mere 17 μm-diameter, but their smaller diameter hampers uniform dispersion within concrete [24]. Yan et al. [25] and Ramujee et al. [26] investigated the mechanical properties of polypropylene fibers. The findings revealed that the ultimate tensile strength of polypropylene fibers ranged from approximately 300 MPa to 700 MPa. Patel et al. [27] conducted an extensive study on UHMWPEF. The research indicated that UHMWPEF experiences fracture under tensile forces ranging from 2000 MPa to 3400 MPa. Therefore, under identical conditions regarding length, diameter, and other factors, polypropylene fibers have a higher probability of fracturing before UHMWPEF when subjected to increased tensile forces. In other words, polypropylene fibers are more susceptible to fracture under the same circumstances. Furthermore, UHMWPEF possesses a significantly elevated molecular weight, thus allowing for the creation of more condensed and uninterrupted molecular layers. It decreases the potential for water molecule infiltration, leading to an enhanced hydrophobic property of UHMWPEF. The hydrophobic nature, firstly, reduces the absorption of free water in concrete by UHMWPEF, thereby partially mitigating its impact on concrete flowability. Secondly, the hydrophobic property assists in maintaining the volume stability of UHMWPEF, preventing any changes in volume due to water absorption or loss. Hence, this further reinforces the adhesion between UHMWPEF and the cementitious matrix. In the research, UHMWPEF, manufactured by Zhejiang Deyan New Materials Company in Hangzhou, China, was chosen as the reinforcing fiber for recycled-brick-aggregate concrete. Apart from its outstanding physical attributes, UHMWPEF exhibits a strong bond with concrete and facilitates co-deformation. This effectively restrains the occurrence of early-stage concrete shrinkage cracks and service period deformation cracks.

#### 2.1.3. Concrete Matrix Material

The cement used in this research is P.O. 42.5 ordinary Portland cement produced by the Asia Tai Group in Harbin, China. Table 4 provides the test results for cement fineness, setting time, and strength properties. The fine aggregate chosen is natural sand with a fineness modulus of 2.5, in compliance with the requirements of the Chinese standard JGJ52-2006 [28].

### 2.2. Experimental Methods

This study involved the preparation of a total of 288 specimens. Among them, 120 specimens were utilized to investigate the influence of RA and UHMWPEF on various properties of concrete. The remaining 168 specimens were allocated for response-surface experiments, providing reliable data support for establishing regression models. Within these 288 specimens, 72 prism specimens measuring 100 × 100 × 515 mm were selected for shrinkage tests; 72 prism specimens measuring 100 × 100 × 400 mm were utilized for flexural strength tests; 72 cube specimens measuring 100 × 100 × 100 mm were designated for chloride ion penetration tests; and 72 prism specimens measuring 100 × 100 × 400 mm were assigned for freeze–thaw cycling tests.

#### 2.2.1. Slump Test

**Figure 3:**
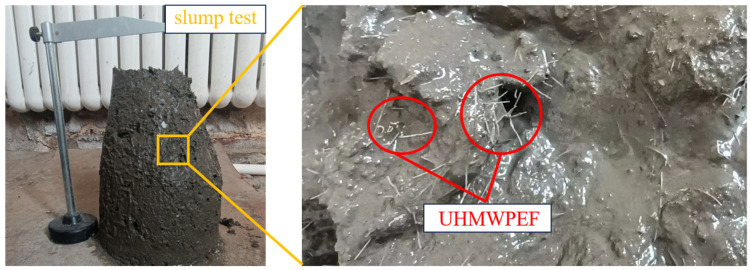
Slump test.

The slump test assesses the workability and water-retaining properties of freshly mixed concrete before setting. An adequate slump is instrumental in enhancing construction quality and the durability of concrete structures. The slump test followed the Chinese standard GB/T 50080-2002 [29], as shown in Figure 3. Each mix proportion underwent three test repetitions, and the arithmetic mean of the three test results was used as the final slump value for that specific mix.

#### 2.2.2. Shrinkage Test

During the concrete curing process, shrinkage occurs, leading to cracks in concrete structures. It has a profound impact on structural safety and durability. Evaluating the shrinkage performance of concrete facilitates the improvement of concrete mix proportions, thus reducing and preventing crack formation. The concrete shrinkage test was conducted by the Chinese standard GB/T 50082-2009 [30].

The specimen dimensions were configured as prisms measuring 100 × 100 × 515 mm. Three specimens were prepared for each mix proportion. The average of the measurements from the three specimens was calculated to determine the shrinkage value for that mix proportion. Throughout the testing period, measurements of specimen length change were recorded at 1 day, 7 days, 14 days, 28 days, 56 days, and 90 days. The concrete shrinkage rate was calculated using Equation (1), where *ε*_0_ represents the concrete shrinkage rate, *L*_0_ denotes the initial length of the specimen, and *L_d_* signifies the specimen length corresponding to the test age.

(1) $$\varepsilon_{d}=\frac{L_{0}-L_{d}}{L_{0}}\times 100\%$$

#### 2.2.3. Flexural Strength Test

The flexural strength test was conducted following the Chinese standard GB/T 50081-2019 [31]. Prism specimens measuring 100 × 100 × 400 mm were selected in the experiments, with three specimens prepared for each group. A universal testing machine with a maximum capacity of 100 KN was employed, and the loading was applied at a uniform rate of 0.05 MPa/s until failure. The average of three trial values (excluding errors not meeting specifications) was selected as the flexural strength for the mix proportion of that group.

#### 2.2.4. Chloride Ion Penetration Test

**Figure 4:**
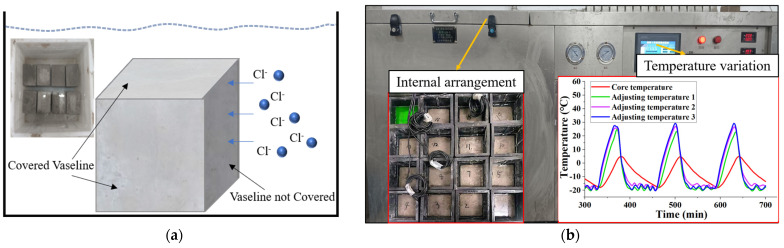
Experimental schematics: (**a**) chloride ion penetration; (**b**) freeze–thaw cycle test.

The chloride ion permeability test was conducted using 100 × 100 × 100 mm cubic specimens. Three specimens were prepared for each mixed proportion group. Specimens aged for 28 days under curing were dried at 110 °C for 24 h. Subsequently, Vaseline was evenly applied to the specimens, leaving only one side untreated. The specimens were then completely immersed in a 3.5% NaCl solution, as depicted in Figure 4a. The side of the specimen without Vaseline coverage was oriented towards the side, as shown in Figure 4a. When the immersion periods reached 14 days, 28 days, 56 days, and 90 days, 5 g of powder was drilled from the side without Vaseline application. The drilling depth was set at 5 mm [32]. The chloride ion concentration in the collected powder was determined according to the method specified in the Chinese standard JGJ/T322-2013 [33].

#### 2.2.5. Freeze–Thaw Cycling Test

**Figure 5:**
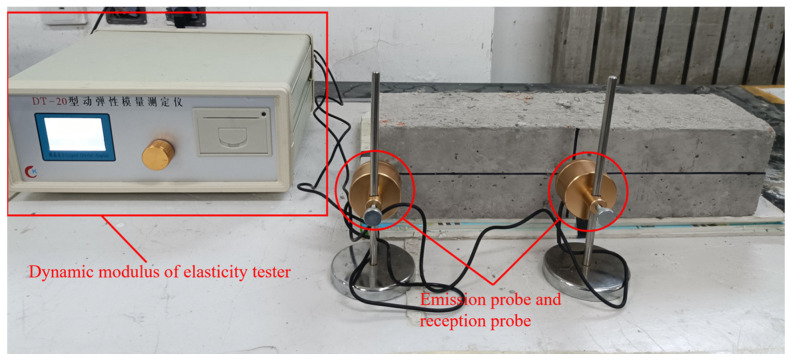
Measuring the relative dynamic elastic modulus of the specimen.

Concrete structures in high-latitude regions inevitably undergo freeze–thaw damage. The freeze–thaw cycling tests in this study were conducted by the GB/T50082-2009 standard [30] using a rapid freezing method. Specimens with dimensions of 100 × 100 × 400 mm were prepared, with three specimens in each group. One hundred sixty-two specimens were prepared for this experiment based on the characteristics of mix proportions from performance tests, response-surface experiments, and freeze–thaw cycling tests. The freeze–thaw testing machine utilized was the KDR-V5, and both freezing and thawing occurred in water, as shown in Figure 4b. Four temperature measurement points were arranged within the freeze–thaw cycling machine to monitor temperature variations throughout the freeze–thaw cycling process. As shown in Figure 4b, the ambient temperature was set between −20 ± 2 °C to 20 ± 2 °C, and the specimen’s central temperature ranged from −18 ± 2 °C to 5 ± 2 °C, meeting the specified temperature range. The duration of a freeze–thaw cycle was set at 3 h, with the thawing time not less than one-third of the entire freeze–thaw cycle time, consistent with the time–temperature variation displayed in the temperature chart. During the freeze–thaw cycling process, monitoring was conducted on the specimens’ relative dynamic modulus of elasticity and flexural strength. The number of freeze–thaw cycles was set at 100. After completing 100 freeze–thaw cycles, the residual flexural strength of the specimens was measured. Additionally, the relative dynamic modulus of elasticity was measured after 25, 50, 75, and 100 freeze–thaw cycles. In this study, the model of the dynamic modulus of the elasticity tester is DT-20 produced by Hua She company in Harbin, China, as illustrated in Figure 5. The measurement process consists of three steps:

1. Determine the contact points between the specimen and the sensor according to the criterion, and apply Vaseline at the contact points to enhance the uniformity of contact;

2. Place the specimen on the testing platform and input the mass and dimensions of the specimen into the instrument;

3. Align the emission probe and the reception probe of the instrument with the contact points, then click the start button to initiate the measurement of the resonant frequencies of the specimen.

The relative dynamic modulus of elasticity was calculated using Formulas (2) and (3). In the equations, *P_i_* represents the relative dynamic modulus of elasticity of the *i*-th specimen after undergoing *n* freeze–thaw cycles, *f_ni_* denotes the lateral resonant frequency of the *i*-th specimen after n freeze–thaw cycles, *f*_0*i*_ is the initial lateral resonant frequency value of the *i*-th specimen before testing, and *P* signifies the relative dynamic modulus of elasticity of a set of recycled-brick-aggregate concrete after n freeze–thaw cycles.

(2) $$P_{i}=\frac{f_{ni}^{2}}{f_{0i}^{2}}\times 100$$

(3) $$P=\frac{1}{3}\sum_{i=1}^{3}P_{i}$$

### 2.3. Experimental Mix Proportions

In order to investigate the influence of RA and UHMWPEF on the concrete properties, the research employed the volume substitution rate of RA for NA and the volume dosage of UHMWPEF as control variables. The volume substitution rates of RA for NA were set at 10%, 20%, 30%, 40%, and 50%. The volume dosages of UHMWPEF were 0.5%, 1.0%, 1.5%, and 2.0%, respectively. Ten different mix proportions were established, and the details for each mix proportion are presented in Table 5. In investigating the impact of UHMWPEF on recycled-brick-aggregate concrete, the replacement rate of RA was designated at 30%. On the one hand, based on preliminary pre-experiments, it was observed that when the RA replacement rate is set at 30%, the concrete can maintain a relative equilibrium among mechanical properties, frost resistance, and others. This approach aims to manifest a reduction in the influence of RA on concrete, mitigating the risk of significant performance variations resulting from excessively low or high RA replacement rates. Researching a foundation of relatively balanced properties provides a more comprehensive demonstration of UHMWPEF’s influence on recycled-brick-aggregate concrete. On the other hand, findings from the research conducted by Meng et al. [3] indicate that exceeding a 30% RA replacement rate results in substantial volumetric deformation during the loading phase of concrete. Consequently, adhering to an RA replacement rate not surpassing 30% is recommended.

## 3. Results and Discussion

### 3.1. Slump

**Figure 6:**
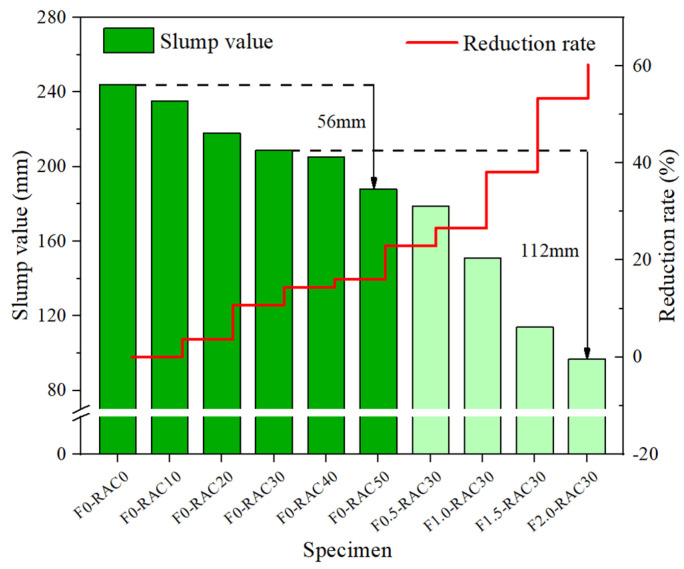
Slump values of concrete with different mix proportions.

**Figure 7:**
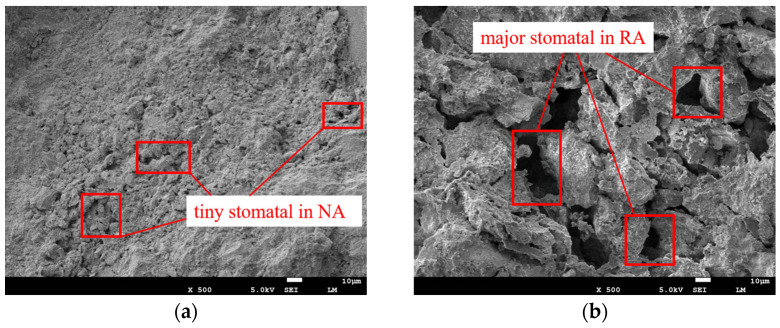
Microscopic pores of different aggregates at SEM with 500×: (**a**) NA; (**b**) RA.

**Figure 8:**
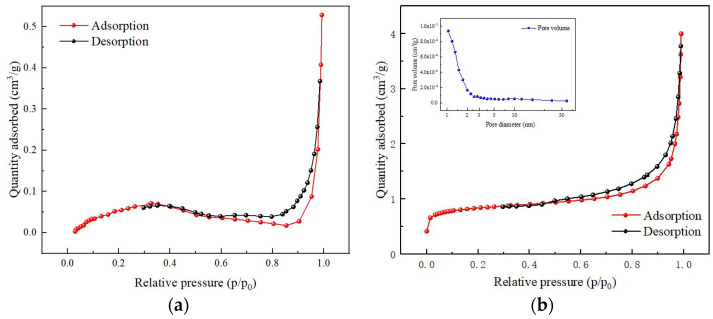
Porosity analysis of different aggregates: (**a**) NA; (**b**) RA.

The slump test can reflect the workability and plasticity of concrete. It directly affects the load-bearing capacity and service life of concrete structures. Figure 6 illustrates the variation in the concrete slump with different RA replacement percentages and UHMWPEF content. As the RA replacement percentage increases, the slump decreases gradually. In the absence of RA, the concrete slump measures 244 mm. However, when the RA replacement percentages reach 10%, 30%, and 50%, the slump decreases by 3.69%, 14.34%, and 22.95%, respectively. It indicates a negative correlation between the concrete slump and the RA replacement percentage. The influence of RA on slump is related to its inherent structure. Figure 7 shows the microstructures of NA and RA under a magnification factor of 500 in SEM. The NA structure exhibits compactness with relatively smaller pores with 10 μm-scale displays. In contrast, the RA structure displays looseness with larger pore sizes. Meng et al. [3] employed X-ray computed tomography (X-CT) to scan RA and recycled-brick-aggregate concrete. The 3D images constructed from the scans revealed the presence of numerous pores and microchannels within the RA. Additionally, Zhu et al. [34] also delved into the pore structure of RA, revealing the existence of numerous capillaries and large pores, which accelerate moisture transport within the RA. Consequently, RA often exhibits a higher water absorption rate under similar conditions, leading to a decreased water–cement ratio within the concrete [35]. Furthermore, the surface properties of RA and natural aggregate (NA) differ. RA exhibits higher roughness and adsorption characteristics, which affect the adhesion between the cement paste and aggregates. The nitrogen adsorption–desorption isotherms for NA and RA are shown in Figure 8. According to the International Union of Pure and Applied Chemistry (IUPAC) classification standards, the adsorption–desorption curve of RA approximates a type IV isotherm. In the nitrogen adsorption test (BET), the magnitude of adsorption reflects the material’s specific surface area [36]. A larger adsorption capacity indicates a larger available adsorption surface, while a smaller capacity suggests fewer available adsorption sites. Compared to NA, the adsorption–desorption curve of RA indicates a larger adsorption capacity, signifying its more complex pore structure and more robust adsorption properties. Additionally, both RA and NA exhibit hysteresis loops in their adsorption–desorption isotherms. The hysteresis loop is a unique phenomenon during the adsorption–desorption process, reflecting the irregularity and complexity of the material’s pore structure. The hysteresis loop is larger and more elongated in RA, indicating larger pores and a better connectivity [37]. This rough structure and these adsorption properties allow cement particles to penetrate the surface of RA more efficiently, with a penetration depth of approximately 120 μm [38]. Consequently, the concrete slump continues to decrease with an increasing replacement rate of RA. Additionally, the presence of dust and impurities in RA also affects the flowability of concrete, further leading to a reduction in its slump.

In addition to the impact of the RA replacement ratio, Figure 6 also illustrates the influence of the UHMWPEF volume fraction on the slump when the RA replacement ratio is 30%. The concrete slump exhibits a more substantial reduction with increased UHMWPEF content. As the UHMWPEF volume fraction increases from 0% to 2%, the slump decreases by 112 mm, reaching a reduction rate of 53.59%. It indicates that UHMWPEF exerts significantly more influence on the slump than RA. The reduction in the concrete slump caused by UHMWPEF is primarily attributed to its alteration of the concrete’s shear strength and viscosity. The fibers in concrete create a three-dimensional network structure [39], which hinders the relative displacement between aggregates and other particles, consequently reducing the workability and plasticity of concrete.

### 3.2. Shrinkage

**Figure 9:**
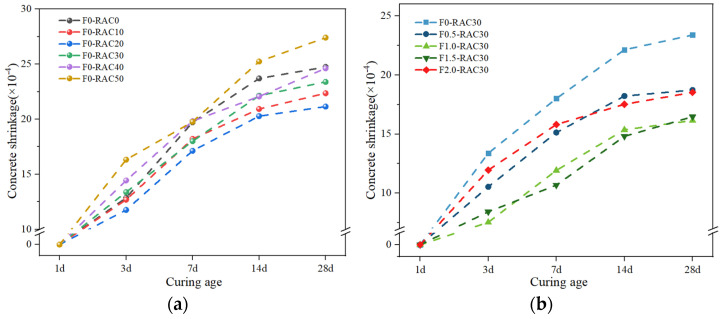
Effects of RA and UHMWPEF on shrinkage performance: (**a**) without UHMWPEF; (**b**) with UHMWPEF.

Excessive concrete shrinkage can lead to structural cracking and deformation, providing pathways for water and other deleterious substances to infiltrate the concrete interior [40]. This reduces the resistance to permeability, frost resistance, and chemical erosion of concrete structures. The influence of RA on concrete shrinkage performance is twofold, as shown in Figure 9a. With an increase in the RA replacement rate, the 28 d concrete shrinkage rate exhibits a changing trend of initial reduction followed by an increase. When the RA replacement rate increases from 0% to 20%, there is a slight reduction in the 28-day concrete shrinkage rate. The shrinkage rate decreases from 24.75 × 10^−4^ to 21.15 × 10^−4^, representing a decrease of 14.54%. However, when the RA replacement rate exceeds 20%, the concrete shrinkage rate increases with the rise in the RA replacement rate. With the RA replacement rate increasing from 20% to 40%, the concrete shrinkage rate has reached a value close to that of F0-RAC30, and this value continues to increase with the rising RA replacement rate. The dual impact of RA on shrinkage performance is associated with its physical properties. When the RA replacement rate is relatively low, it can absorb a portion of the water in the concrete, thereby reducing the initial water–cement ratio of the concrete. A lower water–cement ratio is beneficial in reducing concrete shrinkage. However, the concrete shrinkage rate increases when the RA replacement rate exceeds 20%. Because RA significantly increases the porosity of the concrete at high replacement rates, a high content of RA can increase the porosity of concrete, resulting in accelerated evaporation of moisture from the concrete. On one hand, when moisture moves through tiny capillaries or pores, the capillary or pore walls exert an attractive force on the moisture, creating a negative pressure. This negative capillary pressure causes the cementitious matrix to densify, leading to plastic shrinkage [41]. Thus, as moisture moves within the concrete due to evaporation, this negative pressure exacerbates the concrete’s shrinkage [42,43]. On the other hand, water in concrete exists primarily in the form of free and bound water. It causes a rearrangement of the hydration products within the concrete when free water rapidly evaporates and cannot be replenished in time [44]. These hydration products are gel-like substances formed by the reaction of cement with water, and they act as a binder within the concrete. As the loss of free water triggers a rearrangement of the hydration products in the concrete, it changes the microscopic structure of the concrete [45]. This manifests as volume shrinkage in the concrete at a macroscopic level.

The internal structure and mechanical properties of the concrete can be altered by incorporating UHMWPEF into concrete, thereby improving the shrinkage performance of concrete. Figure 9b illustrates the impact of UHMWPEF on concrete shrinkage performance when the RA replacement rate is 30%. As the UHMWPEF content increases from 0% to 1.0%, the 28-day concrete shrinkage rate decreases by 30.95%. This reduction in shrinkage rate is pronounced compared to a 20% replacement rate of RA. However, when the UHMWPEF content increases to 2.0%, the effectiveness of improving the concrete shrinkage performance diminishes, resulting in an approximate 10.18% reduction in this improvement. It can be explained that a small quantity of fibers contributes to the enhancement of concrete’s crack resistance:

1. The fibers are randomly distributed in the concrete, forming a three-dimensional network structure. This structure provides an inherent support mechanism for concrete [46], alleviating deformations caused by local shrinkage and reducing overall shrinkage rates.

2. When concrete undergoes shrinkage, UHMWPEF can be a bridging agent. Li et al. [47] demonstrated through numerical simulations that the axial stress of the fibers begins to increase as the cement collectively undergoes shrinkage deformation. It indicates that the bridging effect of the fibers can effectively disperse and bear the internal stresses of the concrete, thereby reducing its shrinkage deformation.

3. UHMWPEF can enhance the toughness of concrete, helping to uniformly disperse the strain caused by temperature changes throughout the entire structure. It reduces deformations in different parts of the structure due to temperature variations, thus assisting in reducing overall shrinkage rates.

4. Concrete is a porous material, and its moisture content is influenced by the surrounding environmental humidity. When exposed to low-humidity environments, water loss makes concrete prone to shrinkage. The three-dimensional network structure formed by the fibers in the concrete partially impedes the free movement of water to a certain extent [48]. This barrier effect causes free water within the concrete to traverse the narrow spaces of UHMWPEF when evaporating, thereby slowing down the rate of moisture loss.

**Figure 10:**
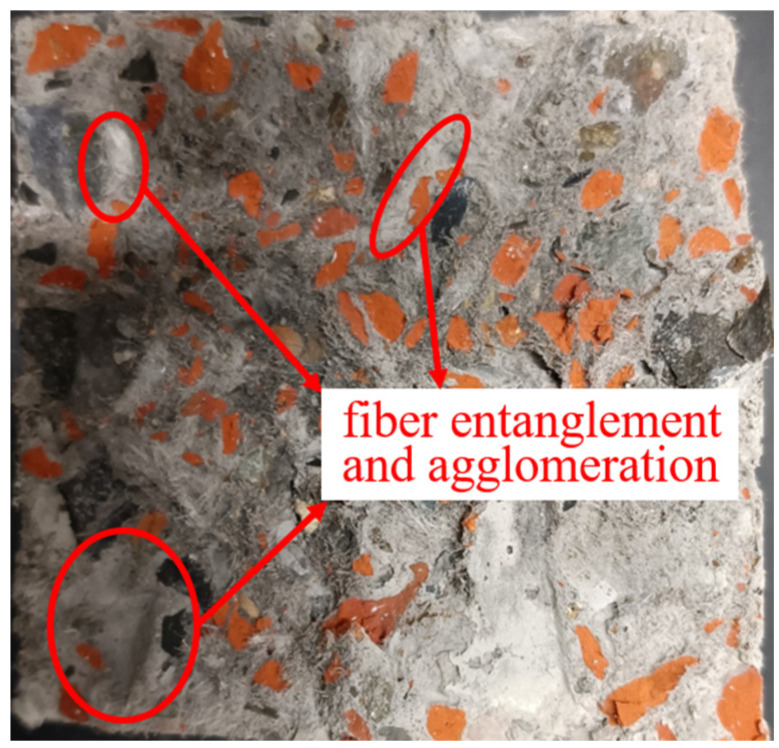
The fiber agglomeration phenomenon occurs in F2-RAC30.

However, when the quantity of UHMWPEF surpasses a certain critical threshold, it can lead to the compaction of the internal concrete network structure or the formation of fiber agglomerations, as illustrated in Figure 10. This results in the creation of voids within the concrete, causing the emergence of relatively large internal gaps and subsequently contributing to concrete shrinkage. On the other hand, an abundance of fibers hampers the progression of hydration reactions. This will result in an extended concrete setting time, causing the concrete to undergo hydration shrinkage over a more prolonged period.

### 3.3. Flexural Strength

**Figure 11:**
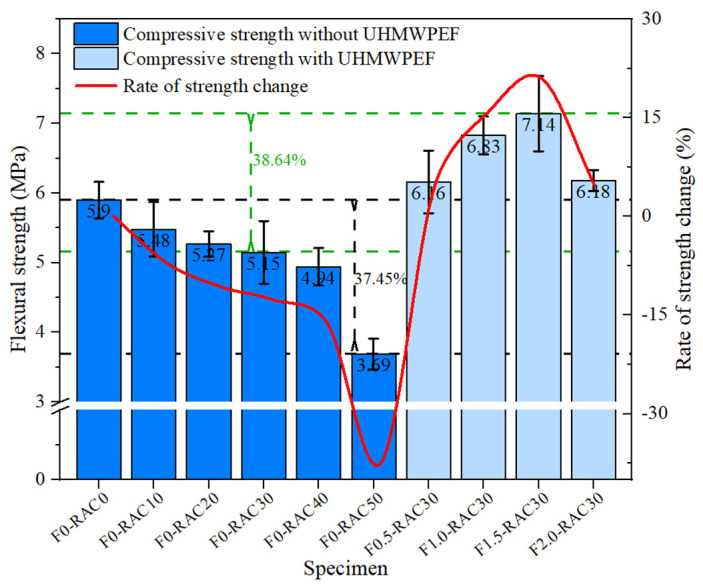
Influence of RA and UHMWPEF on flexural strength.

The type of aggregate can have an impact on the flexural strength of concrete. RA exhibits significantly inferior mechanical properties compared to NA, resulting in a reduction in the flexural strength of concrete. However, introducing UHMWPEF can enhance the mechanical performance of recycled-brick-aggregate concrete. Figure 11 illustrates the influence of RA and UHMWPEF on the flexural performance of concrete. As the RA replacement rate increases from 0% to 40%, the flexural strength of the concrete gradually decreases. For every 10% increase in the RA replacement rate, the flexural strength decreases by approximately 2–7%. This reduction in flexural strength is relatively uniform and moderate. However, when the RA replacement rate increases from 40% to 50%, there is a sudden drop in the flexural strength of concrete. The flexural strength of F0-RAC50 decreases by 21.19% in comparison to F0-RAC40. This decrease is attributed to the inferior mechanical properties of RA. RA is more prone to fracturing than NA when subjected to external forces. RA gradually becomes the dominant component in the concrete as the RA replacement rate reaches 50%. It rapidly increases porosity and weak points within the concrete, substantially reducing the flexural strength at the macro level.

Incorporating UHMWPEF into the concrete effectively mitigates the deteriorating impact of RA on the flexural strength of the concrete. Compared to F0-RAC30, adding 1.5% by volume of UHMWPEF increases the flexural strength of concrete from 5.15 MPa to 7.14 MPa, marking a 38.64% enhancement in flexural performance. This improvement stems from the bridging role played by UHMWPEF within the concrete. When the concrete is subjected to bending loads, UHMWPEF can disperse and absorb some stress. The exceptionally high tensile strength and elastic modulus of UHMWPEF enable it to continue bearing external forces and retard the further propagation of cracks within the concrete after the concrete develops cracks. However, as the content of UHMWPEF increases, it becomes challenging to disperse the fibers in the concrete uniformly, and even fiber agglomeration may occur. This severely hampers the strengthening effect of UHMWPEF on the flexural strength of the recycled-brick-aggregate concrete.

**Figure 12:**
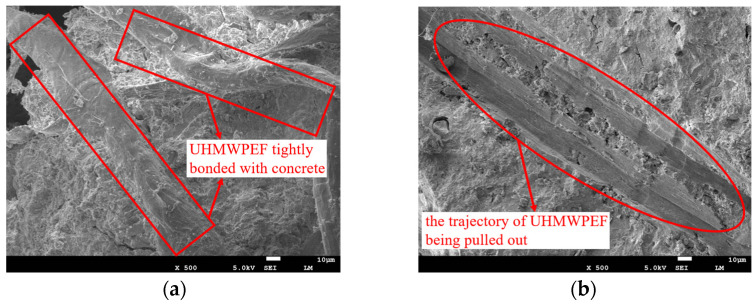
UHMWPEF in concrete: (**a**) intimately adheres to the matrix; (**b**) traces upon being pulled out from the matrix.

**Figure 13:**
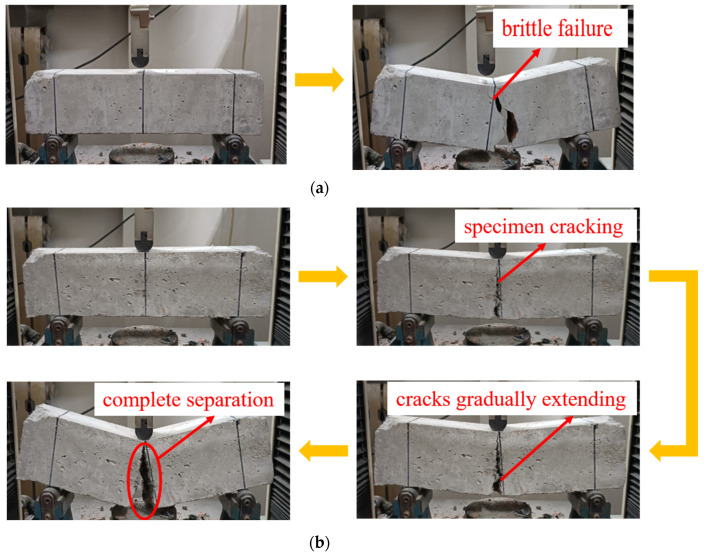
Flexural failure with and without UHMWPEF: (**a**) F0-RAC30; (**b**) F1.5-RAC30.

Figure 12 illustrates the alterations in the microstructure of F1.5-RAC30 induced by UHMWPEF. Figure 12a shows that UHMWPEF forms a strong bond with the concrete matrix, which is a prerequisite for ensuring their synergistic effect. The ultra-high elastic modulus of UHMWPEF, reaching up to 100 GPa, is manifested microscopically as the fibers being drawn out from the concrete matrix rather than being ruptured. Figure 12b illustrates the traces left when UHMWPEF is drawn out from the concrete matrix. This alteration in the microstructure manifests as an increase in the flexural strength and enhancement of ductility of the concrete at the macroscopic level. Figure 13a and b show the conditions of F0-RAC30 and F1.5-RAC30 at the point of failure under bending. F0-RAC30 experiences instantaneous fracture with increased external forces, exhibiting a sudden and rapid failure process. In contrast, the entire failure process of F1.5-RAC30 is notably more gradual. It can be observed that with the increase in external load, F1.5-RAC30 initially develops a crack that propagates from the bottom to the top. The crack gradually enlarges as the external force continues to be applied to the specimen. However, due to the presence of UHMWPEF, the specimen does not fracture entirely, as in the case of F0-RAC30. This indicates that UHMWPEF can enhance the ductility of concrete and effectively suppress the rapid propagation of cracks within the concrete. Ultimately, it transforms the failure mode of concrete from brittle to ductile.

### 3.4. Resistance to Chloride Ion Erosion

**Figure 14:**
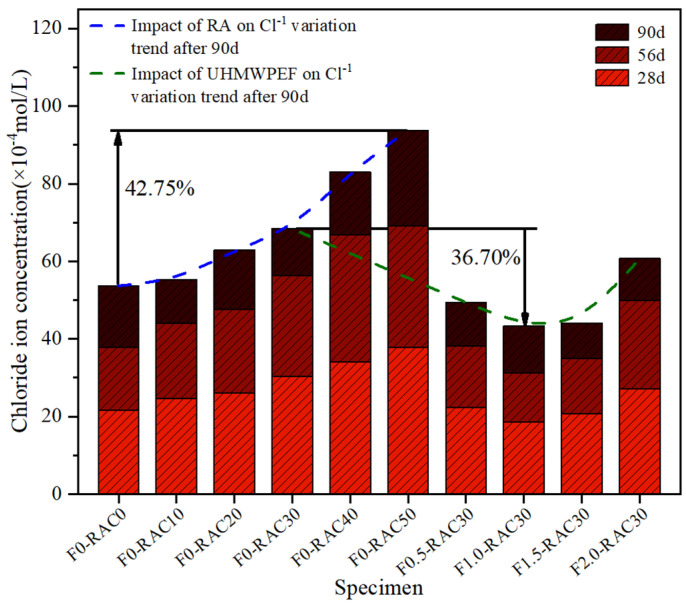
Concrete chloride ion resistance in relation to RA and UHMWPEF.

In order to investigate the influence of RA and UHMWPEF on the concrete’s resistance to chloride ion penetration, the chloride ion concentration within the concrete was tested at 28 days, 56 days, and 90 days of chloride ion exposure, and the results are shown in Figure 14. The extent of chloride ion penetration in the concrete specimens also shows an increasing trend as the RA replacement ratio increases. When the RA replacement ratio increases from 0% to 50%, the chloride ion content within the concrete specimens exposed to chloride ions for 90 d increases from 53.7 × 10^−4^ mol/L to 93.8 × 10^−4^ mol/L, indicating a growth of 42.75%. This variation is associated with the porous structure and adsorption characteristics of RA. Chloride ions primarily ingress into the interior of concrete through penetration and diffusion mechanisms [49]. The high-water absorption characteristics of RA expedite the penetration rate of chloride ions, rendering them more susceptible to ingress into the concrete. Furthermore, RA harbors numerous minuscule pores and fine channels, thus giving rise to the capillary effect [50]. This effect causes water to rise and be retained within the channels, accelerating the transmission of chloride ions throughout the concrete and making it more extensive and rapid.

Comparing the chloride ion concentrations in F0-RAC30, F0.5-RAC30, F1-RAC30, F1.5-RAC30, and F2-RAC30 after 90 days of chloride ion ingress reveals the beneficial influence of UHMWPEF in enhancing the resistance of recycled-brick-aggregate concrete against chloride ion intrusion. Nevertheless, this amelioration diminishes as UHMWPEF surpasses a certain threshold. Among these five sets of specimens, F0-RAC30 exhibited the poorest resistance to chloride ion intrusion, with a chloride ion concentration of 68.4 × 10^−4^ mol/L detected at 90 d. In contrast, the most notable enhancement was observed in the case of F1-RAC30, which demonstrated a 36.7% improvement in its resistance to chloride ion ingress compared to F0-RAC30. The augmentation of resistance against chloride ion erosion can be attributed to two main factors. Firstly, chloride ions primarily infiltrate and diffuse into the concrete interior [49]. However, UHMWPEF can fill inevitable cracks and voids within the concrete, reducing the channels for chloride ion permeation to some extent. Secondly, the three-dimensional network structure formed by fibers within the concrete establishes a physical barrier [48]. The chloride ions must traverse through narrow spaces between the fibers to reach the concrete’s interior during infiltration. This impedes the unrestricted movement of chloride ions within the concrete, consequently decelerating the permeation rate.

### 3.5. Freeze–Thaw Cycling

**Figure 15:**
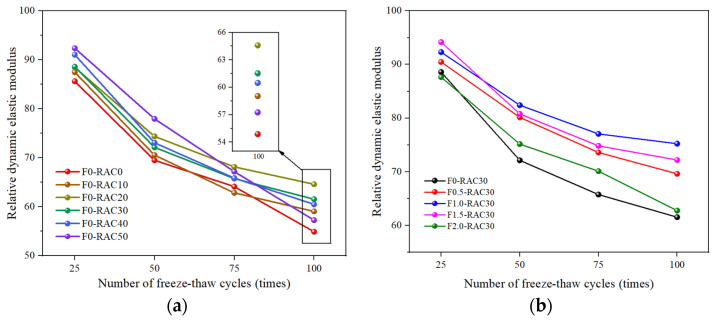
Impact of RA and UHMWPEF on concrete freeze–thaw resistance: (**a**) without UHMWPEF; (**b**) with UHMWPEF.

Freeze–thaw damage is a significant factor leading to the deterioration of concrete structures, especially in high-latitude regions, significantly shortening the lifespan of these structures. Figure 15a illustrates the influence of RA on the freeze–thaw resistance of concrete. Changes in the relative dynamic modulus of elasticity of concrete can reflect the extent of freeze–thaw damage caused by freeze–thaw cycles. Regular concrete’s relative dynamic modulus of elasticity decreases to 54.88 after 100 freeze–thaw cycles. In contrast, the relative dynamic modulus of elasticity for the other five groups of recycled-brick-aggregate concrete remains higher than 54.88. It indicates that RA slows the concrete damage rate during freeze–thaw cycles. During the alternating temperature changes in the freeze–thaw cycle, continuous transformations occur in the states of water and ice. The conversion of water to ice results in volume expansion, generating freeze-induced stress within the concrete when the original pore space cannot accommodate the growth of ice crystals [51]. It leads to freeze–thaw damage when the freeze-induced stress exceeds the internal stress-bearing limit of the concrete. The porous characteristics of aggregates contribute to delaying freeze–thaw damage in concrete. Xia et al. [52] proposed that porous aggregates can accommodate more pore water, thereby enhancing the freeze resistance of concrete. Using numerical simulations, Gong et al. [53] investigated freeze–thaw damage. The results indicated that having more space to accommodate the increased volume due to freeze-induced expansion can alleviate internal stresses within the material. It is well-known that air entrainment enhances the freeze resistance of concrete [54]. Air-entraining agents generate bubbles within the concrete, dispersed throughout as a buffer. When the concrete is subjected to freeze–thaw damage, these bubbles absorb the stresses caused by water expansion, slowing the formation and propagation of internal cracks. Scanning electron microscopy experiments revealed that RA exhibits more pores than NA. Nitrogen adsorption experiments confirmed that the pore volume of RA is significantly larger than that of NA. Therefore, as a porous material, RA plays a role similar to air bubbles in concrete.

Furthermore, the porous characteristics of RA enable it to accommodate more air. Upon incorporation into concrete, additional air is introduced with RA. This portion of gas helps to mitigate damage caused by freeze–thaw cycles to some extent. On the other hand, the porous nature results in a lower thermal conductivity of RA [55]. This property allows RA to slow down the heat exchange rate in concrete. Under freeze–thaw cycle conditions, rapid temperature changes induce uneven expansion and contraction within the concrete, increasing internal stresses. RA can decelerate the rate of temperature change within the concrete [56], alleviating the internal stresses and strains caused by temperature gradients and contributing to maintaining the concrete’s relative stability.

Fibers can establish a three-dimensional network structure within recycled-brick-aggregate concrete, thereby enhancing the continuity and ductility of the concrete. Consequently, the frost resistance of concrete is improved by adding UHMWPEF to concrete, which aids in dispersing freeze–thaw-induced stresses caused by water expansion. However, excessive UHMWPEF can lead to clustering and void formation within the concrete, detrimental to its frost resistance. Figure 15b illustrates the relationship between the UHMWPEF content and the frost resistance of concrete under a 30% RA replacement rate. The relative dynamic modulus of elasticity of concrete exhibits an initial rise followed by a decline with an increase in fiber substitution rates. Concrete maintains a relatively high relative dynamic modulus of elasticity even after 100 freeze–thaw cycles when the UHMWPEF content reaches 1.0%. However, the relative dynamic modulus of elasticity decreases when the UHMWPEF content surpasses 1.0%. This implies that an optimal UHMWPEF content exists that can maximize the frost resistance of recycled brick-aggregate concrete.

**Figure 16:**
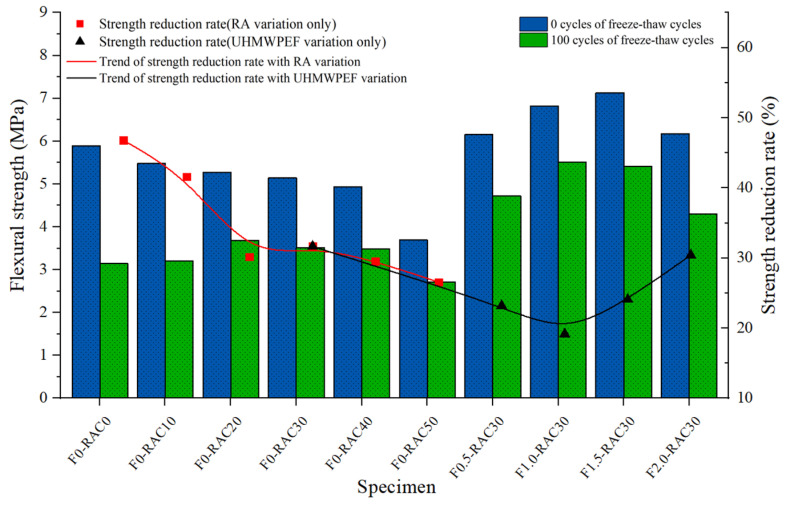
Impact of freeze–thaw cycles on the flexural performance of F-RAC.

Figure 16 illustrates specimens’ flexural strength variation after 0 and 100 freeze–thaw cycles. The reduction in strength of the concrete after 100 freeze–thaw cycles, relative to its initial strength, exhibits a decreasing trend with the increase in the replacement ratio of RA. It indicates that RA can mitigate the damage caused to concrete by freeze–thaw cycles. Within a specific range, this mitigating effect improves with an increase in the replacement ratio of RA. This enhancement is intricately linked to RA’s porous structure and low thermal conductivity. However, an excessive RA content leads to a rapid decline in the initial flexural performance of concrete. Adding UHMWPEF as a reinforcing material in concrete effectively enhances the initial flexural strength of recycled-brick-aggregate concrete. As the volume fraction of UHMWPEF increases from 0.0% to 1.0%, there is a noticeable improvement in the initial flexural strength of the specimens. Simultaneously, the freeze–thaw resistance of the specimens is enhanced with the incorporation of UHMWPEF. The three-dimensional mesh structure formed by UHMWPEF in concrete effectively shares internal stresses induced by freezing expansion, contributing to the volumetric stability of the concrete. Additionally, the bridging effect of UHMWPEF effectively inhibits the initiation and propagation of microcracks in the concrete, thereby slowing down the damage caused by the freeze–thaw process.

## 4. Response-Surface Optimization

The response-surface methodology is an effective model for analyzing the coupling effects of various factors on the target with multiple responses. It employs function values at specific points to approximate absolute values by establishing nonlinear functions [57]. The underlying principle involves designing experimental plans for different combinations of variables and subsequently obtaining response values corresponding to each set of parameters in the experimental design. It utilizes a multivariate quadratic regression equation to model the functional relationship between the independent variables and the response values. Ultimately, the optimal process parameters are sought through an analysis of the regression equation. In response-surface analysis, the response value can be expressed as Equation (4), where *f*(*x*_1_, *x*_2_, ···, *x_n_*,) is a function of the independent variables *x*_1_, *x*_2_, ···, *x_n_*, and *k* is the error term. The purpose of response-surface methodology experimental design is to achieve the optimal value of ŷ = *f*(*x*_1_, *x*_2_, ···, *x_n_*,) + *k* by selecting suitable values for the independent variables *x*_1_, *x*_2_, ···, *x_n_*.

(4) $$Y=f(x_{1},x_{2},\cdot \cdot \cdot ,x_{n})+k$$

### 4.1. Response-Surface Design

There exist distinct relationships between various concrete properties, the RA replacement rate, and the content of UHMWPEF. Therefore, the utilization of response-surface design for optimizing the combination of the RA replacement rate and UHMWPEF content can significantly enhance the F-RAC performance. This research employed a central composite design (CCD) within the response-surface methodology, with the volume replacement rate of RA and the volume content of UHMWPEF as independent variables. Utilizing Minitab software 19, a CCD design was employed to determine the ratios of RA and UHMWPE, resulting in 14 experimental combinations. Among these, the experiments at the vertices were conducted four times (No. 1–No. 4), those at the center points were carried out six times (No. 5–No. 7 and No. 12–No. 14), and experiments at the axial points were performed four times (No. 8–No. 11). The response variables encompassed the 28-day shrinkage of F-RAC, flexural strength, chloride ion concentration at 90 days, and the relative dynamic modulus after 100 freeze–thaw cycles. The design scheme and experimental outcomes of the CCD tests for RA and UHMWPE are presented in Table 6.

### 4.2. Variance Analysis of the Regression Model

The variance analysis of the regression models was conducted based on the results of the response-surface experiments, leading to the establishment of the following four sets of regression models. Here, *A* denotes the substitution rate of RA, measured in %, while *B* represents the volume fraction of UHMWPEF, also expressed in %.

(5) $$Shrinkage=22.56-0.2047\times A-9.96\times B+6.145\times 10^{-3}\times A^{2}+4.077\times B^{2}-6.5\times 10^{-3}\times A\times B$$

(6) $$Flexural\;strength=5.563+3.6\times 10^{-3}\times A+3.138\times B-7.41\times 10^{-4}\times A^{2}-1.269\times B^{2}+1.7\times 10^{-4}\times A\times B$$

(7) $$\begin{array}{c} Chloride\;ion\;concentration= \end{array}55.30-0.06\times A-40.45\times B+0.01807\times A^{2}+22.63\times B^{2}-0.317\times A\times B$$

(8) $$Relative\;dynamic\;elastic\;modulus=58.46+0.315\times A+25.49\times B-6.16\times 10^{-3}\times A^{2}-11.38\times B^{2}-0.0472\times A\times B$$

A variance analysis was conducted in Equations (5)–(8) to verify the reliability of the regression models. The results of the variance analysis are presented in Table 7. A larger F-value and a smaller *p*-value indicate the model’s significance [58]. If the *p*-value is less than 0.05, it indicates the significance of the model. In this research, the four regression models established all had *p*-values within 0.05, confirming their significance. The coefficient of determination, R^2^, is typically used to measure the goodness of fit of a regression model to experimental results. R^2^ varies between 0 and 1, representing the extent to which the regression model can explain the variability or fluctuations in the dependent variable [59]. A higher R^2^ value indicates the model’s ability to explain the variation in the dependent variable better, while a lower R^2^ value suggests the model’s poorer explanatory power. The R^2^ values for Equations (5)–(8) are all above 0.95, indicating a good fit between the models and the experimental results. Pre-R^2^, representing the predictive coefficient of determination, is a statistical metric used to assess the predictive performance of a regression model [60]. It can evaluate the ability of the model to predict new data. Similar to R^2^, the closer Pre-R^2^ is to 1, the stronger the model’s predictive capability on new data. The Pre-R^2^ values for the four regression models established in this research are 0.8154, 0.8159, 0.8466, and 0.7945, indicating that the models possess good predictive capabilities.

### 4.3. Response-Surface and Contour Analysis

**Figure 17:**
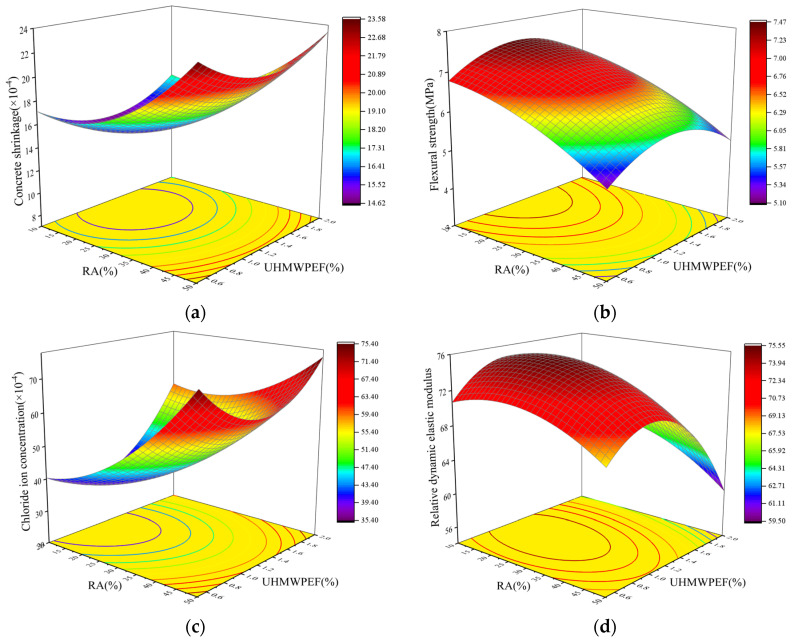
A 3D plot of factor interactions: (**a**) shrinkage; (**b**) flexural strength; (**c**) chloride ion resistance; (**d**) freeze–thaw resistance.

The response-surface plot visually demonstrates the degree to which the interaction of the two factors affects the response variable. A more pronounced surface curvature indicates a more substantial influence of the factor interaction on the response variable. Conversely, a flatter surface suggests a weaker influence. The response-surface plots for the impact of the RA replacement rate and UHMWPEF content on concrete properties, as determined based on the regression model established in Section 4.2, are depicted in Figure 17. With RA replacement rates ranging from 10% to 50% and UHMWPEF content varying from 0.5% to 2.0%, the 28 d shrinkage of F-RAC fluctuates within the range of 14.62 × 10^−4^ to 23.58 × 10^−4^. Under the same conditions, the variations in F-RAC’s flexural strength, 90 d chloride ion concentration, and relative dynamic modulus after 100 freeze–thaw cycles are in the ranges of [2.10, 7.47] MPa, [35.40, 75.40] × 10^−4^ mol/L, and [59.5, 75.55], respectively. By examining the response-surface plots, it can be ascertained that the order of surface curvature from high to low is as follows: Figure 17d > Figure 17c > Figure 17b > Figure 17a. This indicates that the interaction between RA and UHMWPEF has the most significant influence on the freeze–thaw resistance of F-RAC. In contrast, the interaction has the most negligible impact on the shrinkage performance.

**Figure 18:**
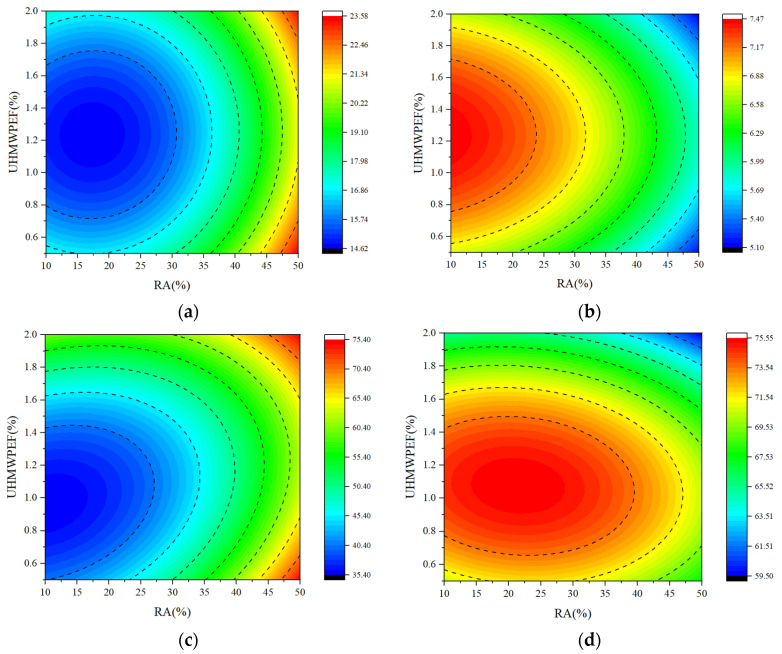
Contour plot of factor interactions: (**a**) shrinkage; (**b**) flexural strength; (**c**) chloride ion resistance; (**d**) freeze–thaw resistance.

Figure 18 illustrates the contour plots of the interaction between RA and UHMWPEF for the four sets of models. By comparing the number of contour lines in the X and Y directions, the relative influence on the concrete properties due to RA and UHMWPEF can be determined [61]. Figure 18 depicts the contour plots of the interaction between RA and UHMWPEF for the four sets of models. The relative impact on concrete properties due to RA and UHMWPEF can be ascertained by comparing the number of contour lines in the X and Y directions. Regarding concrete shrinkage, flexural strength, and resistance to chloride ion erosion, it can be observed that the contour lines in the X-axis direction are more densely distributed. This implies that for these three performance indicators, the influence of the RA replacement ratio on F-RAC is more significant than that of UHMWPEF content. It is evident that the contour lines in the Y-axis direction are more densely packed in the contour plots than in the X-axis direction when examining the freeze–thaw resistance of F-RAC. This indicates that the UHMWPEF content more significantly influences the freeze–thaw resistance of F-RAC. Furthermore, the shape of the contour lines can also reflect the interaction between the two variables. When the contour lines take on an elliptical shape, it suggests a more pronounced interaction between the two variables. The two variables signify a weaker interaction if the contour lines are closer to circular, aligning with the response surfaces’ curvature.

### 4.4. Multi-Objective Optimization

Various relationships exist between the different properties of F-RAC, the substitution rates of RA, and the addition of UHMWPEF, with some correlations being completely opposite. Therefore, the optimization of the regression models using the desirability function is employed to prepare an F-RAC that can simultaneously meet various performance requirements. Three optimization objectives were established in the research, and the results are presented in Table 8. The first optimization objective is simultaneously achieving the minimum shrinkage and the maximum flexural strength. Under this condition, the 28-day shrinkage of F-RAC is 14.76 × 10^−4^, and the flexural strength is 7.43 MPa. The substitution rate of RA is 12.57%, and the volume fraction of UHMWPEF is 1.24%. The second optimization objective, building upon the first optimization, aims to maximize concrete’s resistance to chloride ion erosion as much as possible. Optimization based on this criterion reveals that the optimization objective can be achieved when the substitution rate of RA and the volume fraction of UHMWPEF are 12% and 1.15%, respectively. Under these conditions, the F-RAC exhibits a 28-day shrinkage of 14.82 × 10^−4^, a flexural strength of 7.43 MPa, and a 90 d chloride ion concentration of 36.24 × 10^−4^ mol/L.

The third optimization objective considers the frequent occurrence of concrete structural damage due to freeze–thaw cycles in high-latitude regions. Therefore, the F-RAC design should prioritize maximizing freeze–thaw resistance while meeting relevant mechanical performance indicators. In this scenario, the optimization objective is to simultaneously achieve low shrinkage, high flexural strength, outstanding resistance to chloride ion erosion, and freeze–thaw durability, all while considering the prerequisite of mechanical performance. This optimization objective can be achieved when the RA replacement rate is 14.02% and the UHMWPEF content is 1.13%. The results indicate that the 28-day shrinkage, flexural strength, and 90 d chloride ion concentration are 14.73 × 10^−4^, 7.4 MPa, and 36.24 × 10^−4^ mol/L, respectively. At this point, the fitted value for the relative dynamic modulus of F-RAC after 100 freeze–thaw cycles is 75.19.

Experiments corresponding to the RA replacement rate and UHMWPEF volume fraction for the third optimization scenario were conducted. The experimental results and fitted values are presented in Table 9. The optimized F-RAC’s average shrinkage was 15.38 × 10^−4^, which was 4.41% higher than the predicted value. The average actual flexural strength and chloride ion concentration were 7.26 MPa and 38.58 × 10^−4^ mol/L, respectively, falling within the confidence intervals of (7.2099, 7.5831) and (32.27, 40.10), respectively. After 100 cycles of freeze–thaw testing, the measured relative dynamic modulus of elasticity for F-RAC was 71.61, which was 3.58 lower than the predicted value. While the experimental value was close to the lower limit of the 95% confidence interval, it remained within an acceptable range.

The root mean square error (RMSE) is a statistical metric to quantify the disparities between model-predicted and actual observed values. A smaller RMSE indicates more accurate predictions, while a larger RMSE suggests more significant prediction errors in the model [62]. Table 10 delineates the RMSE of the model based on the third optimization scenario. For the shrinkage performance, flexural strength, resistance to chloride ion erosion, and resistance to freeze–thaw cycling, the model’s RMSE values are 0.98, 0.34, 2.96, and 5.14, respectively. Due to variations in experimental factors, direct comparisons between RMSE values are not feasible. Normalizing the RMSE by dividing it by the corresponding model-predicted values yields 0.0665, 0.0459, 0.0818, and 0.0684. This signifies minimal differences between the model’s predicted and actual experimental values, affirming the reliability of the optimization results for F-RAC based on the response-surface method. Furthermore, the model exhibits the most stable predictive capability for the flexural strength of F-RAC, closely followed by predictions for shrinkage performance and frost resistance based on the RMSE/fitting value in Table 9. The model’s predictive ability for chloride ion erosion resistance in F-RAC demonstrates relatively more significant fluctuation. Nevertheless, the model demonstrates a high degree of fitting with experimental values.

## 5. Conclusions

This research investigated the influence of the recycled-brick-aggregates (RA) replacement ratio for natural aggregates and the ultra-high molecular weight polyethylene fiber (UHMWPEF) content on concrete. The research objectives encompass shrinkage, flexural strength, resistance to chloride ion penetration, and freeze–thaw resistance. The impact mechanisms of RA and UHMWPEF on concrete were explored by analyzing both macroscopic properties and microscopic structure. The interactions between RA and UHMWPEF on concrete were analyzed using response-surface methodology. Simultaneously, utilizing regression models, a multi-objective optimization of concrete was conducted to determine the optimal combination of RA and UHMWPEF. In conclusion, the following findings are derived:

(1) Both RA and UHMWPEF have a detrimental impact on the concrete slump, with the effect of UHMWPEF being pronounced. The coarse surface of RA and its high water absorption rate reduce the actual water–cement ratio of concrete. Furthermore, the formation of a three-dimensional network structure by UHMWPEF in concrete hinders the relative displacement between aggregates and other particles.

(2) Excessive RA significantly diminishes the concrete’s resistance to shrinkage, flexural strength, and resistance to chloride ion erosion. However, this effect is modulated by UHMWPEF. The ultra-high molecular weight of UHMWPEF provides it with an exceptionally high elastic modulus, enabling it to share a portion of the stress within the concrete effectively. With a fixed RA replacement rate, UHMWPEF has a dual impact on concrete performance. Within a specific range of volume fractions, the addition of UHMWPEF enhances concrete performance. However, the beneficial effects decrease rapidly beyond a certain threshold and may even hurt concrete.

(3) The bridging effect of UHMWPEF and the porous structure of RA play pivotal roles in the freeze–thaw resistance of concrete. RA provides additional space to accommodate the increase in volume when water transforms into ice. The bridging effect of UHMWPEF contributes to sharing temperature-induced stress and resisting crack propagation, thereby preserving the overall integrity of the concrete.

(4) The impact of the interaction between RA and UHMWPEF on concrete performance was analyzed using response-surface methodology. It was determined that the order of influence on concrete properties due to the interaction between RA and UHMWPEF, from high to low, is as follows: freeze–thaw resistance > resistance to chloride ion erosion > flexural strength > shrinkage resistance.

(5) Three optimization objectives were proposed to determine the optimal proportions of RA and UHMWPEF. When the replacement rate of RA is 14.02%, and the content of UHMWPEF is 1.13%, the concrete achieves the best shrinkage resistance, flexural strength, chloride ion erosion resistance, and freeze–thaw resistance. Experimental results demonstrate a high level of agreement between predicted values and actual measurements, indicating the reliability of the optimization results for F-RAC based on the response-surface methodology.

## Figures and Tables

**Table 1:** Chemical composition of RA.

Chemical Compositions	SiO_2_ (%)	Al_2_O_3_ (%)	Fe_2_O_3_ (%)	K_2_O (%)	MgO (%)	Others (%)
Content	62.72	19.83	6.62	2.49	1.95	6.39

**Table 2:** Physical properties of RA and NA.

Aggregate Types	Apparent Density/Kg·m^−3^	Bulk Density/Kg·m^−3^	Water Absorption/%	Crush Value/%
Recycled-brick aggregate	2130	1206	18.3	24.3
Natural aggregate	2794	1542	1.2	4.8

**Table 3:** Physical properties of different fibers.

Fiber	Appearance	Density /(g/cm^3^)	Diameter /μm	Tensile Strength /MPa	Elastic Modulus /GPa	Elongation /%
UHMWPEF	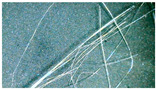	0.97	40	2000–3400	100	2.8
Polypropylene fiber	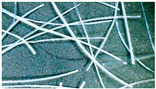	0.91	100	300–700	5.8	19.9
Basalt fiber	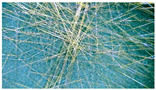	2.6	17	2000	85	3.1
Steel fiber	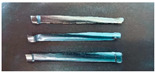	7.8	300–800	1270	200	3.4–4

**Table 4:** Cement quality inspection results.

Test Items	Fineness/%	Setting Time/(min)		Compressive Strength/(MPa)		Flexural Strength/(MPa)	
		Initial Setting Time	Final Setting Time	3 Day	28 Day	3 Day	28 Day
Standard value	≤10.0	≥45	≤600	≥17	≥42.5	≥3.5	≥6.5
Detection value	3.9	155	370	28.5	51.4	5.1	8.1

**Table 5:** Mix designs of each group (Kg/m^3^).

Label	Cement	5–10 mmNA	10–20 mmNA	5–10 mmRA	10–20 mmRA	Sand	Water	Fiber
F0-RAC0	487	341.4	796.6	0.0	0.0	613	233	0
F0-RAC10	487	307.3	716.9	26.0	60.7	613	233	0
F0-RAC20	487	273.1	637.3	52.1	121.5	613	233	0
F0-RAC30	487	239.0	557.6	78.1	182.2	613	233	0
F0-RAC40	487	204.8	478.0	104.1	242.9	613	233	0
F0-RAC50	487	170.7	398.3	130.1	303.6	613	233	0
F0.5-RAC30	487	239.0	557.6	78.1	182.2	613	233	4.9
F1-RAC30	487	239.0	557.6	78.1	182.2	613	233	9.7
F1.5-RAC30	487	239.0	557.6	78.1	182.2	613	233	14.6
F2-RAC30	487	239.0	557.6	78.1	182.2	613	233	19.4

Note: Rn-RACm represents the concrete with a volume dosage of UHMWPEF at n% and a volume substitution rate of RA for NA at m%. For instance, F1.5-RAC30 denotes concrete with a 1.5% volume dosage of UHMWPEF and a 30% volume substitution rate of RA for NA.

**Table 6:** Response-surface design and results.

Serial Number	RA (%)	UHMWPEF (%)	Shrinkage (×10^−4^)	Flexural Strength (MPa)	Chloride Ion Concentration (×10^−4^ mol/L)	Relative Dynamic Elastic Modulus
1	10	0.5	17.19	6.74	42	70.34
2	50	0.5	24.51	5.23	77	66.25
3	10	2	17.82	6.45	65	63.61
4	50	2	24.75	4.95	81	56.69
5	30	1.25	15.84	7.02	43	75.66
6	30	1.25	15.48	6.93	41	71.06
7	30	1.25	15.57	6.85	47	74.05
8	1.72	1.25	16.05	7.69	36	73.88
9	58.28	1.25	23.61	5.22	71	68.77
10	30	0.19	19.74	5.48	60	66.97
11	30	2.31	19.26	5.76	69	59.94
12	30	1.25	15.75	7.11	40	74.73
13	30	1.25	15.39	6.95	39	75.14
14	30	1.25	15.66	6.84	46	77.39

**Table 7:** Analysis of variance for the regression model.

Source	F-Value	*p*-Value	R^2^	Pre-R^2^	Statistical Significance
Equation (5)	50.55	<0.0001	0.9774	0.8154	Significant
Equation (6)	50.70	<0.0001	0.9775	0.8159	Significant
Equation (7)	37.69	<0.0001	0.9700	0.8466	Significant
Equation (8)	25.05	<0.0001	0.9555	0.7945	Significant

**Table 8:** Multi-objective optimization.

Target	Factor		Predicted Values			
	RA (%)	UHMWPEF (%)	Shrinkage (×10^−4^)	Flexural Strength (MPa)	Chloride Ion Concentration (×10^−4^ mol/L)	Relative Dynamic Elastic Modulus
Min. S; Max. F.	12.57	1.24	14.76	7.43	—	—
Min. S and C; Max. F.	12.00	1.15	14.82	7.43	36.24	—
Min. S and C; Max. F and R.	14.02	1.13	14.73	7.40	36.18	75.19

Note: S denotes the 28-day shrinkage; F represents the flexural strength; C indicates the 90-day chloride ion concentration, and R signifies the relative dynamic modulus after 100 freeze–thaw cycles.

**Table 9:** Verification of optimization results in the third case.

Response	Average Actual Value	Fitting Value	Residual	95% Confidence Interval
Shrinkage (×10^−4^)	15.38	14.73	0.65	(13.974, 15.494)
Flexural strength (MPa)	7.26	7.40	−0.14	(7.2099, 7.5831)
Chloride ion concentration (×10^−4^ mol/L)	38.58	36.18	2.4	(32.27, 40.10)
Relative dynamic elastic modulus	71.61	75.19	−3.58	(70.527, 79.848)

**Table 10:** Model statistical analysis based on the third situation.

Item	Three Experimental Values			Variance (S^2^)	Root Mean Square Error (RMSE)	RMSE/Fitting Value
Shrinkage (×10^−4^)	14.35	15.77	16.02	0.96	0.98	0.0665
Flexural strength (MPa)	7.57	6.84	7.37	0.11	0.34	0.0459
Chloride ion concentration (×10^−4^ mol/L)	40.36	36.24	39.14	8.75	2.96	0.0818
Relative dynamic elastic modulus	70.94	76.42	67.47	26.39	5.14	0.0684

## Data Availability

Data are contained within the article.
